# Palmer’s Instruments

**Published:** 1874-01

**Authors:** 


### PALMER'S INTSRUMENTS.

We have had for some months past in use a set of Dr. Corydon Palmer's filling instruments. In attempting to describe them it would be impossible to compare them to any that had preceded them, except to say that they embody about all that is good in all that have hitherto been produced, and discard the impracticable and worthless.

The workmanship excels anything that has hitherto been produced; it is as nearly perfect as anything that we can imagine. They come up to and even excel anything we had conceived of, during the many years past in which we have been pleading for finer and better instrnments. It would be in vain for us to attempt to describe them in detail; the only way to know what they are, is to procure them and use them; then, and not till then, can any one know them. No one who desires to have the best thing can afford to be without them. It has been objected "that they are too expensive." In reply, we would say that there is more work and skill displayed upon them than upon any instruments we have ever seen, to the same amount. They are manufactured by Dr. S. S. White, under the special direction of Dr. Palmer, which is a sufficient guarantee of quality. We presume they can be obtained through any of the dental depots.

